# Both 3,5-Diiodo-L-Thyronine and 3,5,3′-Triiodo-L-Thyronine Prevent Short-term Hepatic Lipid Accumulation via Distinct Mechanisms in Rats Being Fed a High-Fat Diet

**DOI:** 10.3389/fphys.2017.00706

**Published:** 2017-09-14

**Authors:** Rosalba Senese, Federica Cioffi, Pieter de Lange, Cristina Leanza, Liliana F. Iannucci, Elena Silvestri, Maria Moreno, Assunta Lombardi, Fernando Goglia, Antonia Lanni

**Affiliations:** ^1^Dipartimento di Scienze e Tecnologie Ambientali, Biologiche e Farmaceutiche, Università degli Studi della Campania, “Luigi Vanvitelli” Caserta Caserta, Italy; ^2^Dipartimento di Scienze e Tecnologie, Università degli Studi del Sannio Benevento, Italy; ^3^Dipartimento di Biologia, Università degli Studi di Napoli Federico II Naples, Italy

**Keywords:** thyroid hormones receptors, thyroid hormones, thyroid hormone response element, lipogenesis, lipid metabolism

## Abstract

3,3′,5-triiodo-L-thyronine (T3) improves hepatic lipid accumulation by increasing lipid catabolism but it also increases lipogenesis, which at first glance appears contradictory. Recent studies have shown that 3,5-diiodothyronine (T2), a natural thyroid hormone derivative, also has the capacity to stimulate hepatic lipid catabolism, however, little is known about its possible effects on lipogenic gene expression. Because genes classically involved in hepatic lipogenesis such as SPOT14, acetyl-CoA-carboxylase (ACC), and fatty acid synthase (FAS) contain thyroid hormone response elements (TREs), we studied their transcriptional regulation, focusing on TRE-mediated effects of T3 compared to T2 in rats receiving high-fat diet (HFD) for 1 week. HFD rats showed a marked lipid accumulation in the liver, which was significantly reduced upon simultaneous administration of either T3 or T2 with the diet. When administered to HFD rats, T2, in contrast with T3, markedly downregulated the expression of the above-mentioned genes. T2 downregulated expression of the transcription factors carbohydrate-response element-binding protein (ChREBP) and sterol regulatory element binding protein-1c (SREBP-1c) involved in activation of transcription of these genes, which explains the suppressed expression of their target genes involved in lipogenesis. T3, however, did not repress expression of the TRE-containing ChREBP gene but repressed SREBP-1c expression. Despite suppression of SREBP-1c expression by T3 (which can be explained by the presence of nTRE in its promoter), the target genes were not suppressed, but normalized to HFD reference levels or even upregulated (ACC), partly due to the presence of TREs on the promoters of these genes and partly to the lack of suppression of ChREBP. Thus, T2 and T3 probably act by different molecular mechanisms to achieve inhibition of hepatic lipid accumulation.

## Introduction

Lipogenesis, lipolysis, and carbohydrate metabolism are the principal pathways that modulate metabolism. In these pathways, hepatic metabolism plays a key role, since the liver is the major site of triglyceride synthesis, lipogenesis, carbohydrate metabolism, glycolysis, and glycogen synthesis (Dentin et al., 2005, 2006). Lipogenesis is regulated through acute control of key enzymes that are subject to gene transcriptional regulation (Dentin et al., 2005; Desvergne et al., 2006), post-translational modification (e.g., phosphorylation), and allosteric modulation of their activities in response to dietary status and hormone levels (Uyeda and Repa, 2006). An increased hepatic lipogenesis/lipolysis ratio and increased lipolysis in adipocytes could lead to deposition of lipids in the liver leading to the development of nonalcoholic fatty liver disease (NAFLD). NAFLD is emerging as the most common liver disease associated with insulin resistance and obesity that can evolve into steatohepatitis, cirrhosis, and hepatocellular carcinoma (Bugianesi et al., 2002; Zafrani, 2004). The thyroid hormone, 3,3′,5-triiodo-L-thyronine (T3), modulates lipid metabolism in the liver and promotes both lipid catabolism and lipogenesis. Indeed, T3 has long been recognized as an important inducer of hepatic lipogenic enzyme gene transcription (Roncari and Murthy, 1975; Llobera et al., 1979; Oppenheimer et al., 1987, 1991; Freake et al., 1989; Cable et al., 2009; Zhu and Cheng, 2010; Yao et al., 2014; Chen et al., 2015) by binding to specific nuclear receptors, thyroid hormone receptors (TRs) that are present in the liver and also widely distributed throughout the body (Yen, 2001; Brent, 2012; Sinha and Yen, 2014). By activating TRs, in particular the β isoform (TRβ), T3 elicits an important effect on lipid metabolism, as well as on metabolic rate. Due to its ability to increase energy expenditure and improve lipid profile, the potential use of T3 as hypolipidemic agent has been proposed. However, T3 may cause a clinical thyrotoxic state, characterized primarily by heart failure and skeletal muscle wasting. Recently, research has focused on identifying and developing thyroid hormone (TH) analogs that are both tissue- and TRβ- (the isoform predominantly involved in TH-induced hepatic metabolism) (Moreno et al., 2008) selective in order to stimulate hepatic metabolism without causing side effects particularly on the heart (Raparti et al., 2013).

Previous studies have shown that 3,5-diiodo-L-thyronine (T2), a natural TH derivative, could stimulate metabolism both in humans and rodents (Moreno et al., 1997; Lanni et al., 1998; Antonelli et al., 2011; Grasselli et al., 2012) and also rapidly affect mitochondrial bioenergetic parameters (Lombardi et al., 2007, 2009; Mollica et al., 2009; Cavallo et al., 2013, 2016; Grasselli et al., 2014). In rats, these effects were not associated with side effects usually caused by T3. In animals receiving a high-fat diet (HFD), T2 prevented both adiposity and body weight-gain by increasing lipid mobilization and hepatic beta-oxidation (Lanni et al., 2005; de Lange et al., 2011; Moreno et al., 2011; Grasselli et al., 2012), accompanied by increased expression of peroxisome proliferator-activated receptor α (PPARα) (de Lange et al., 2011). Moreover, *in vitro*, it has been shown that both T2 and T3 were able to reduce excess fat storage and to mobilize triglyceride from lipid droplets in primary cultures of rat hepatocytes overloaded with lipids (Grasselli et al., 2011, 2015). In contrast to T3, T2 also decreased hepatic lipogenesis in HFD-fed rats (de Lange et al., 2011). T3 and T2 thus appear to act on HFD-induced hepatic lipogenesis in opposite ways.

The aim of this study was to evaluate and compare possible transcriptional effects of both T2 and T3 using a model of hepatic lipid accumulation. To this end, we assessed the effects of both T2 and T3 on the expression of genes containing thyroid hormone response elements (TREs) such as, acetyl-CoA carboxylase (ACC) (Yin et al., 2002), SPOT 14 (Liu and Towle, 1994), and fatty acid synthase (FAS) (Radenne et al., 2008) that are involved in hepatic lipogenesis. ACC controls the conversion of acetyl-CoA to malonyl-CoA during fatty acid (FA) synthesis. Subsequently, FAS generates palmitic acid (C16:0) utilizing acetyl-CoA and malonyl-CoA. SPOT 14 is a well-known transcriptional target of thyroid hormone (Brown et al., 1997), and acts as a transcription factor associated with increased hepatic triglyceride accumulation and enhanced lipogenesis (Wu et al., 2013). An important transcription factor involved in the regulation of expression of *ACC, FAS*, and *SPOT 14* is sterol regulatory element binding protein-1c (SREBP-1c) (for review see: Berlanga et al., 2014). T2 and T3 modulate *SREBP-1c* expression and activity through various mechanisms (Hashimoto et al., 2006; de Lange et al., 2011; Rochira et al., 2013). *ACC, FAS*, and *SPOT 14* are in turn targeted by carbohydrate-response element-binding protein (ChREBP), a major factor that controls the activation of glucose-induced lipogenesis in the liver. The expression of *ChREBP* is stimulated by T3 in a TRβ-dependent manner (Hashimoto et al., 2009; Gauthier et al., 2010). We have chosen to study the anti-lipidemic effects of T2 and T3 using a 1-week period of HFD feeding, leading to lipid accumulation but not to a possible irreversible hepatosteatosis. We chose the dose of T2 [25 μg/100 g body weight (BW)] because in our previous studies, this dose effectively prevented hepatic lipid accumulation without undesirable side effects, and is typically 10-fold lower than the doses used in other studies where cardiac side effects were observed (Padron et al., 2014; Jonas et al., 2015; Moreno et al., 2016). We chose a 10-fold lower dose of T3 (2.5 μg/100 g BW) because it is known that at this dose, T3 activates transcription through TRs/TREs without thyrotoxic effects (Senese et al., 2014).

## Materials and methods

### Animals and animal care

All animals received humane care according to the criteria outlined in the Guide for the Care and Use of Laboratory Animals prepared by the National Academy of Sciences and published by the National Institutes of Health. All animal protocols were approved by the Committee on the Ethics of Animal Experiments of the University of Napoli Federico II (Italy) and the Italian Minister of Health (Permit Number: 2011/0041469). Every effort was made to minimize animal pain and suffering. Male Wistar rats (250–300 g, aged 8 weeks) were kept one per cage in a temperature-controlled room at 28°C (thermoneutrality for rats) under a 12-h light/12-h dark cycle. Before commencement of the study, a commercial mash (Charles River Laboratories, Calco, Italy) was available *ad libitum* and the animals had free access to water. At the start of the study (day 0), and after 7 days of acclimatization to thermoneutrality, the rats were divided into four groups of five and treated for 1 week as follows.

- The first group (N) received a standard diet (total metabolizable percentage of energy: 60.4 carbohydrates, 29 proteins, 10.6 fat J J^−1^; 15.88 kJ gross energy g^−1^; Muscedola, Milan, Italy) with a daily injection intraperitoneally of vehicle. These rats were only used to show the effect of the HFD on hepatic lipid accumulation and not as a control vs. HFD-, HFD+T3-, and HFD+T2-animals.
- The second group (HFD) received a high-fat diet [280 g diet supplemented with 395 g of lyophilized lamb meat (Liomellin, Milan, Italy), 120 g cellulose (Sigma-Aldrich, St. Louis, MO, USA), 20 g mineral mix (ICN Biomedical, Solon, OH, USA), 7 g vitamin mix (ICN), and 200 g low-salt butter (Lurpak, Denmark); total metabolizable percentage of energy: 21 carbohydrates, 29 proteins, 50 fat J J^−1^; 19.85 kJ gross energy g^−1^] with a daily injection intraperitoneally of vehicle. The HFD rats represent the control group in comparison to HFD+T3 and HFD+T2 groups to study the effects of T3/T2.
- The third group (HFD+T2) received the same HFD as in the second group with a daily injection of T2 (25 μg 100 g^−1^ BW intraperitoneally) (Sigma-Aldrich).
- The fourth group (HFD+T3) received the same HFD as in the second group with a daily injection of T3 (2.5 μg 100 g^−1^ BW intraperitoneally).

All animals continued to have free access to water. At the end of the treatment, the rats were anesthetized by intraperitoneal injection of chloral hydrate (40 mg 100 g^−1^ BW) and then decapitated. Liver, heart, gastrocnemius muscle, and abdominal white adipose tissues were excised, weighed, and immediately frozen in liquid nitrogen and stored at −80°C for later processing.

### Staining of hepatic lipid droplets

Sections of livers from five animals in each experimental group were fixed in formol-calcium, and 10-μm frozen sections were subsequently stained with Sudan black B for the detection of fat according to standard procedures.

### Liver triglyceride estimation

Liver triglycerides content was determined using an Infinity kit (Sigma-Aldrich) following the instruction of the producer.

### RNA isolation

Total liver RNA was isolated using the TRIZOL standard protocol (Invitrogen Life Technologies, Milano, Italy). Tissue/TRIZOL mixtures were homogenized using a polytron, keeping the viscosity of the solution to a minimum to ensure effective inactivation of endogenous RNAse activity. First-strand RNA was treated with DNase I, Amp Grade, to remove residual genomic DNA (Invitrogen Life Technologies).

### Quantitative real-time PCR

Total RNA (1 μg) was used to generate cDNA strands in a 20-μl-reaction volume using the Super Script First Strand Synthesis System for RT-PCR (Invitrogen). An equivalent of 25 ng total cDNA was subsequently used in the amplification. Real-time quantitative RT-PCR (QRT-PCR) was carried out with 50 nM gene-specific primers and IQ SYBR Green supermix (BioRad) using standard cycle parameters on a MyiQ2 (BioRad). A melting curve analysis was completed following amplification from 55 to 95°C to assure product identification and homogeneity. Each sample was repeated in triplicate and normalized to the housekeeping gene, β-actin, to compensate for any differences in starting quantity of total RNA.

PCR primers were designed using the Primer 3 program (Untergasser et al., 2012), and then synthesized and verified by sequencing at Eurofins Genomics (Ebersberg, Germany). Primers used were the following.

βACT sense 5′-GCTACAGCTTCACCACCACA-3′
βACT antisense 5′-AGGGCAACATAGCACAGCTT-3′
SREBP-1c sense 5′-GGCCCTGTGTGTACTGGTCT-3′
SREBP-1c antisense 5′-AGCATCAGAGGGAGTGAGGA-3′
ACC1 sense 5′-GACGTTCGCCATAACCAAGT-3′
ACC1 antisense 5′-CTGCAGGTTCTCAATGCAAA-3′
FAS sense 5′-GGATGTCAACAAGCCCAAGT-3′
FAS antisense 5′-CAGAGGAGAAGGCCACAAAG-3′
SPOT 14 sense 5′-CTGAGGAAGACAGGCTTTCG-3′
SPOT 14 antisense 5′-TTCTGGGTCAGGTGGGTAAG-3′
CHREBP sense 5′-CAGGATGCAGTCCCTGAAAT-3′
CHREBP antisense 5′-GAGGTGGCCTAGGTGGTGTA-3′
TRB sense 5′-AGCAGGCTGACTTGGAATGT-3′
TRB antisense 5′-GGTTACAGAAGGCCATGGAA-3′
D1 sense 5′-AGACTGGAAGACAGGGCTGA-3′
D1 antisense 5′-GCCTTGAATGAAATCCCAGA-3′.

### Serum TT4 and TT3 measurements

Serum total T4 (TT4) and total T3 (TT3) were measured using commercially available immunoassay kits (materials and protocols were supplied by Byk-Sangtec Diagnostica, Germany).

### Statistical analysis

Data are expressed as mean ± SEM and are normally distributed. The statistical significance of differences between the HFD, HFD+T2, and HFD+T3 groups (*n* = 5) was determined using one-way analysis of variance (ANOVA) followed by Tukey's test. Differences were considered significant at *P* < 0.05.

## Results

### Organ weight and adiposity in N, HFD, and HFD+iodothyronines groups

To verify whether administration of iodothyronines to HFD animals might alter organ weight and induce a thyrotoxic-like state, we measured the weights of organs such as liver, heart, skeletal muscle, and adipose tissue that are most sensitive to thyroid status. The administration of both iodothyronines did not alter the weight of the heart. Both T2 and T3 significantly reduced the weight of subcutaneous adipose tissue and liver. Only T3 slightly, but significantly reduced the gastrocnemius muscle weight. Serum TT4 and TT3 levels did not change between HFD and HFD+T2 animals, whereas levels of TT3 significantly increased in the HFD+T3 compared to HFD and HFD+T2 groups (Table 1).

**Table 1:** Organ weights and total T4 (TT4) and T3 (TT3) serum levels from N, HFD, HFD+T2, and HFD+T3 rats after 1 week of treatment.

	**Organ weight (g)**						**Serum levels (nM)**	
**1 week**	**Heart**	**Liver**	**WAT visc**	**WAT subc**	**Gastrocn**	**BAT**	**TT4**	**TT3**
N	1.15 ± 0.06	12.8 ± 0.041	9.83 ± 0.81	5.10 ± 0.30	2.05 ± 0.08	0.35 ± 0.02	66 ± 3.2	0.80 ± 0.05
HFD	1.21 ± 0.09	13.8 ± 0.07^$^	11.21 ± 0.98	6.20 ± 0.48^*^	2.30 ± 0.09	0.33 ± 0.02	74 ± 4.2	0.84 ± 0.04
HFD+T2	1.13 ± 0.08	12.9 ± 0.06^#^	9.89 ± 0.84	4.77 ± 0.31^#^	2.03 ± 0.07	0.37 ± 0.04	69 ± 3.4	0.75 ± 0.04
HFD+T3	1.15 ± 0.10	12.1 ± 0.06^**^	9.40 ± 0.79	4.10 ± 0.25^#^	1.90 ± 0.11^#^	0.4 ± 0.05	61 ± 2.5	1.52 ± 0.13^**^

*Values represent the mean ± SEM for five rats*.

* P < 0.05 vs. N;

# P < 0.05 vs. HFD;

$ P < 0.05 vs. N, HFD+T2, and HFD+T3;

** *P < 0.05 vs. N, HFD, HFD+T2*.

### Appearance and lipid accumulation in the livers

**Figure 1:**
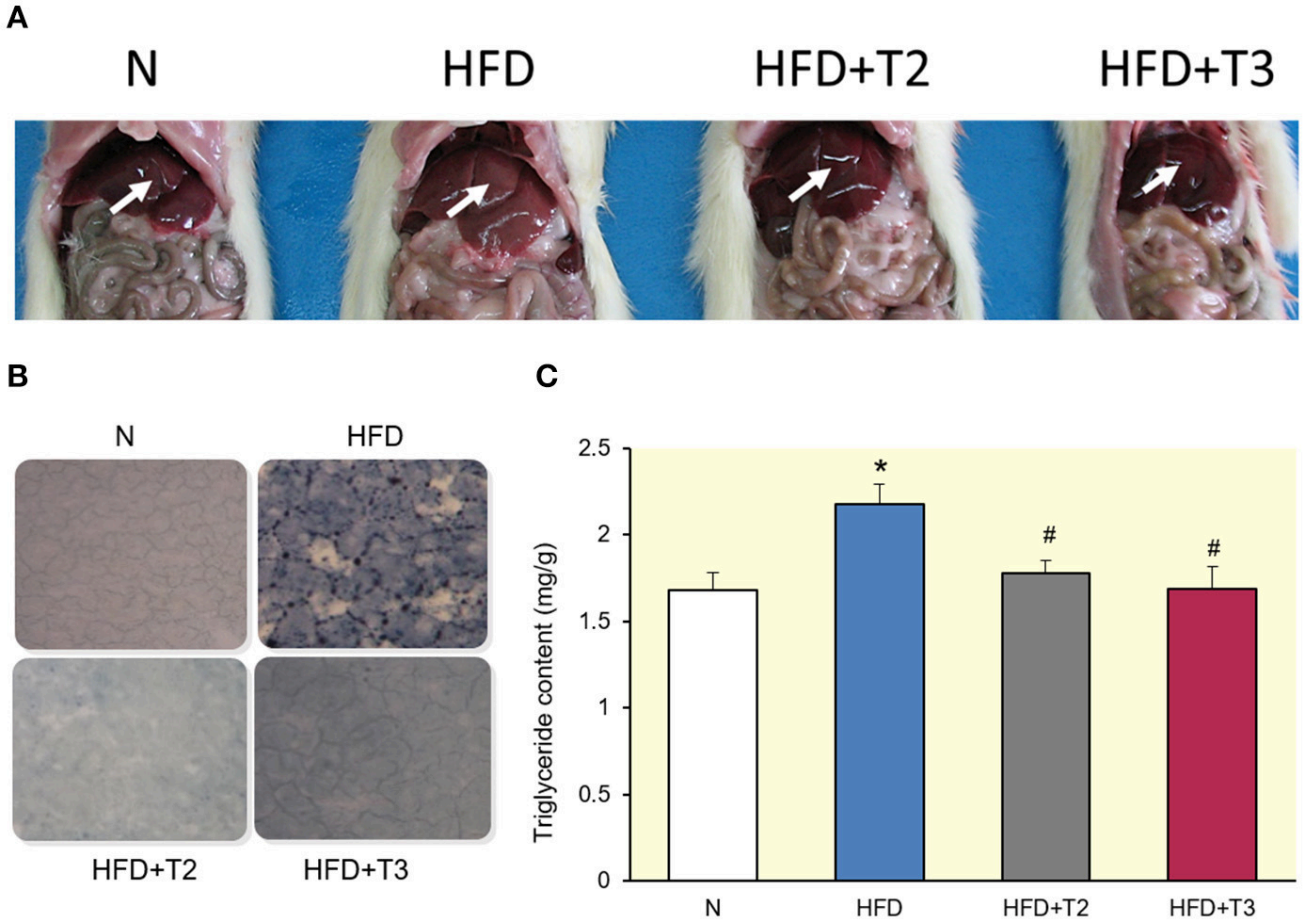
Comparison between livers of rats fed a standard diet (N) or a high-fat diet (HFD), untreated or treated for 1 week with T2 (HFD+T2) or T3 (HFD+T3). **(A)** Macroscopic view (arrows highlighting liver colors). **(B)** Histology. **(C)** Histograms showing liver triglyceride (TG) content. Values represent the mean ± SEM for five rats. ^*^P < 0.05 vs. N, ^#^*P* < 0.05 vs. HFD.

One week of HFD feeding revealed a clear difference in appearance of the livers, with the HFD livers looking pale with respect to the other conditions (Figure 1A). Sudan black staining of liver sections revealed lipid accumulation in the livers of rats fed on HFD compared to those fed a standard diet (N) (Figure 1B). Of interest, the HFD animals receiving iodothyronines were protected from fat accumulation in the liver (Figure 1B). In addition, triglyceride content was higher in HFD compared to N rats, and returned to normal when treated with either T2 or T3 (Figure 1C).

### Effects of T2 and T3 on hepatic lipogenic genes

**Figure 2:**
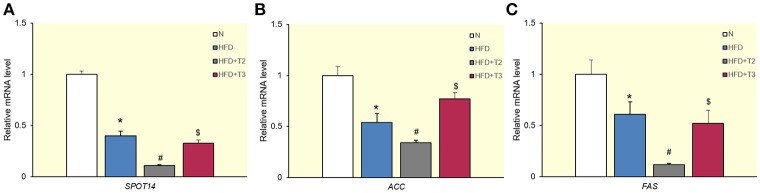
Effects of T2 and T3 on *SPOT 14* **(A)**, *acetyl-CoA carboxylase (ACC)* **(B)**, and *fatty acid synthase (FAS)* **(C)** gene expression in rats fed a high-fat diet for 1 week. Values represent the mean ± SEM for five rats used in quantitative RT-PCR analysis. Expression was normalized to β-actin. **(A)** ^*^P < 0.05 vs. N; ^#^*P* < 0.05 vs. all conditions; ^$^*P* < 0.05 vs. N and HFD+T2. **(B)** ^*^P < 0.05 vs. N; ^#^*P* < 0.05 vs. all conditions; ^$^*P* < 0.05 vs. HFD and HFD+T2. **(C)** ^*^P < 0.05 vs. N; ^#^*P* < 0.05 vs. all conditions; ^$^*P* < 0.05 vs. N and HFD+T2.

Following evaluation of the effects exerted by iodothyronines on lipid accumulation, we next examined their effects on lipogenic genes. We first evaluated the effects of both iodothyronines on well-known lipogenic genes, such as *SPOT 14* (Figure 2A), *ACC* (Figure 2B), and *FAS* (Figure 2C) that contain TRE. When either T3 or T2 was administered to HFD animals, T3 significantly enhanced the expression of *ACC* (Figure 2B) but was ineffective on the other two genes. T2, on the other hand, significantly reduced the expression of all considered lipogenic genes.

### Effects of T2 and T3 on expression of SREBP-1c and ChREBP transcription factors

**Figure 3:**
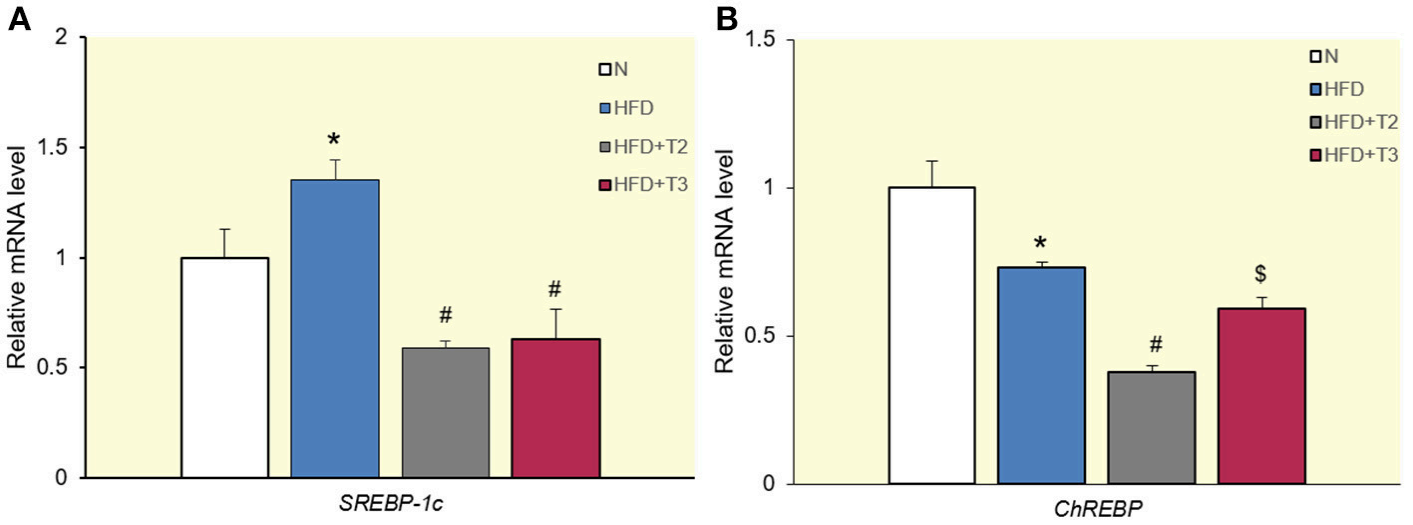
Effects of T2 and T3 on *sterol regulatory element binding protein-1c (SREBP-1c)* **(A)** and *carbohydrate-response element-binding protein (ChREBP)* **(B)** gene expression in rats fed a high-fat diet for 1 week. Values represent the mean ± SEM for five rats used in quantitative RT-PCR analysis. Expression was normalized to β-actin. **(A)** ^*^P < 0.05 vs. N; ^#^*P* < 0.05 vs. N and HFD. **(B)** ^*^P < 0.05 vs. N; ^#^*P* < 0.05 vs. all conditions; ^$^*P* < 0.05 vs. N and HFD+T2.

The expression data reveal a significantly higher *SREBP-1c* expression in HFD than HFD+T2 and HFD+T3 rats (Figure 3A). This implies that SREBP-1c does not mediate the modulation effects of T3 on the expression of *SPOT14, FAS*, and *ACC*. To verify whether ChREBP played a functional role in the upregulation of *these genes* by T3, we measured mRNA levels for this gene in the various treatment subgroups. The injection of T2 to HFD animals significantly reduced the expression of *ChREBP1* compared to HFD and HFD+T3 animals (Figure 3B). The fact that T3 did not significantly lower *ChREBP1* expression compared to HFD may be due to the fact that the promoter of this gene contains a functional TRE (see Table 2 and references therein).

**Table 2:** Presence of positive and negative thyroid hormone response elements of the genes studied.

**Genes lipogenesis**	**Positive and negative TREs**
	**References**
ChREBP (+TRE)	Hashimoto et al., 2009
SREBP-1c (-TRE)	Hashimoto et al., 2006
SPOT 14 (+TRE)	Liu and Towle, 1994
ACC (+TRE)	Yin et al., 2002
FAS (+TRE)	Radenne et al., 2008
DIO1(+TRE)	Toyoda et al., 1995
TRβ (+TRE)	Suzuki et al., 1994

### Effects of T2 and T3 on the expression of deiodinase type I and TRβ

**Figure 4:**
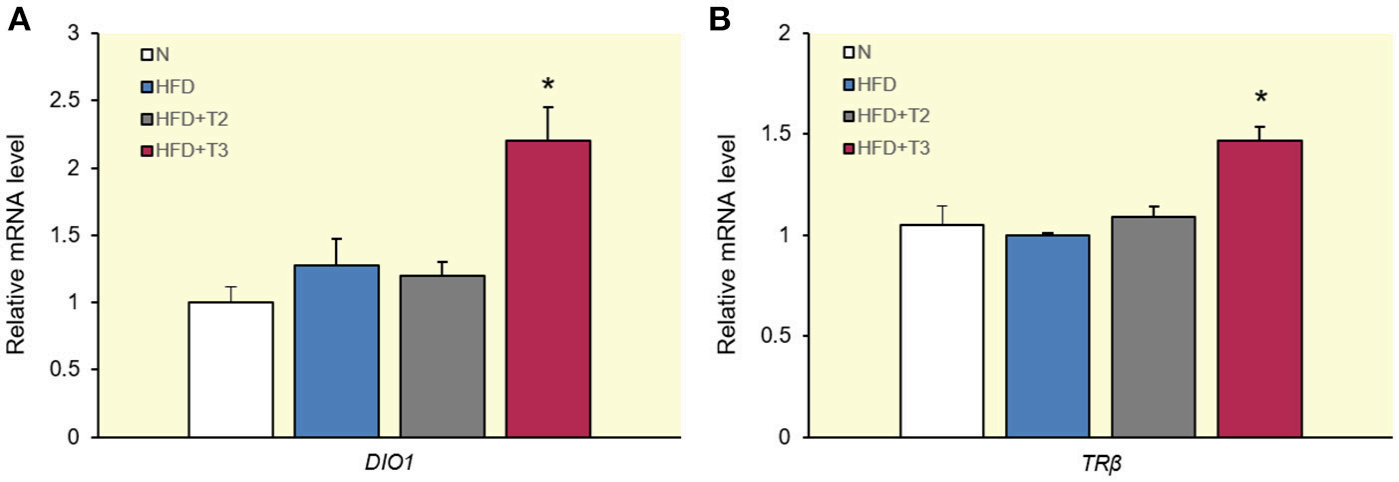
Effects of T2 and T3 on *deiodinase type 1 (DIO1)* **(A)** and *TR*β **(B)** expression in rats fed a high-fat diet for 1 week. Values represent the mean ± SEM for five rats used in quantitative RT-PCR analysis. Expression was normalized to β-actin. **(A)** ^*^P < 0.05 vs. N, HFD, and HFD+T2. **(B)** ^*^P < 0.05 vs. N, HFD, and HFD+T2.

To verify that the differential TRE-mediated regulation of gene expression with the given doses of T2 and T3 in livers after 1 week of HFD feeding did not only apply to genes involved in lipogenesis, we also evaluated the expression of TRE-containing genes, which are considered classical markers of the transcriptional effects of T3, such as *deiodinase type I* (*DIO1*) (Toyoda et al., 1995) (Figure 4A) and *TR*β in humans (Suzuki et al., 1994) and tilapia fish (Hernandez-Puga et al., 2016) (Figure 4B). As shown in Figure 4, and underlining the results obtained in this study, T3 upregulated the expression of these genes with respect to HFD, whereas T2 failed to do so.

## Discussion

To our knowledge, the present study is the first that used animals receiving HFD to study the transcriptional effects of T2 and T3 on genes possessing TRE that are primarily involved in hepatic lipogenesis. Hepatic *de novo* lipogenesis is mainly regulated by transcriptional control of lipogenic gene expression by transcription factors such as SREBP-1c and ChREBP. Iodothyronines play a role in these mechanisms (Hashimoto et al., 2006, 2009; Gauthier et al., 2010). The regulation of lipid accumulation by TH is complex. Recently, it has been observed that hypothyroidism is closely associated with NAFLD (Chung et al., 2012), and that thyrotropin increases hepatic triglyceride content through upregulation of SREBP-1c activity (Yan et al., 2014).

Based on the data reported here, T2 and T3 administration to HFD-rats for 1 week reduced hepatic lipid deposits, as shown by liver fat accumulation and triglyceride content (Figure 1).

T3 administration to HFD animals resulted in an inhibition of *SREBP-1c* expression, but paradoxically upregulated *ACC* expression and did not downregulate *SPOT 14* and *FAS*. Hence, T3 does not appear to reduce HFD-associated lipid accumulation through its effects on these target genes. This same paradox has been suggested in a previous work in which it was observed that *SREBP-1c* expression was downregulated by T3 (Cable et al., 2009), despite the known stimulative effect of T3 on expression of lipogenic genes that possess TREs (Liu and Towle, 1994; Yin et al., 2002; Radenne et al., 2008). This finding was suggested to be one of the reasons for the relatively weak anti-steatotic effects of T3 in the longer term (Cable et al., 2009). The effects of T3 on SREBP-1c and its target genes are due to the fact that their promoters respond to SREBP-1c as well as TRs (Cable et al., 2009; Cavallo et al., 2016). Thus, although the reduction of *SREBP-1c* expression by T3 should result in downregulation of the *SREBP-1c* target genes, this effect is prevented due to presence of TREs in their promoters. The downregulation of *SREBP-1c* by T3, in turn, is likely due to the presence of negative TREs in its promoter (Hashimoto et al., 2006) (see Table 2). With regard to T2, the expression of genes involved in lipogenesis tightly followed the decreased expression of *SREBP-1c* and *ChREBP*, suggesting that T2 does not stimulate the transcription of these genes through their TREs. We have previously shown that T2 reduces *SREBP-1c* expression in liver by SIRT1-mediated deacetylation and inactivation of the SREBP-1c protein. Since this protein acts as a transcription factor inducing its own transcription, SREBP-1c inactivation results in reduced *SREBP-1c* transcription (de Lange et al., 2011).

Because the promoter of *ChREBP* contains a TRE (Hashimoto et al., 2009) (see Table 2), it is perhaps not surprising that HFD+T3 animals had higher *ChREBP* mRNA levels that were not significantly different from HFD animals, but significantly so with respect to HFD+T2 animals in which *ChREBP* expression was reduced with respect to HFD animals (Figure 3). This implies that during a 1-week HFD treatment, the upregulation of *ChREBP* and *SREBP-1c* common target genes by T3 with respect to T2 may thus be in part ChREBP-mediated, and may not only be a direct TRE-mediated effect on the target genes themselves. Together, these results, show that T3 works by directly transcribing key genes involved in lipogenesis through TR/TREs, which can either be positive or negative.

The gene encoding the TR mainly involved in hepatic lipid metabolism, *TR*β, has been shown to contain TREs in humans (Suzuki et al., 1994) and tilapia fish (Hernandez-Puga et al., 2016). Interestingly, the human *TR*β promoter was transactivated by T3 (Katz and Koenig, 1994) whereas the fish *TR*β promoter was transrepressed by T2. We observed a transactivating effect of T3 on rat *TR*β in the HFD context, but T2 at the given dose, did not alter *TR*β expression.

The effects of T2 unlike T3, on lipid accumulation may be mediated by other pathways without directly altering transcription of genes harboring positive or negative TREs. Indeed, on the basis of previous studies, it has been shown that the affinity of T2 for *TR*β is 500-fold less than that of T3 (Ball et al., 1997; Mendoza et al., 2013), and that induction of TRE-mediated transcription requires much higher concentrations of T2 than T3. We here verified that this was indeed confirmed *in vivo*. The action of T2 is likely due to post-translational modifications. In fact, we have previously shown that T2, under the same experimental conditions activated deacetylase sirtuin 1 (SIRT1) (de Lange et al., 2011), a member of the sirtuin family in the liver. SIRT1 has emerged as a key metabolic sensor for regulating metabolic homeostasis. Using rats fed a HFD for 4 weeks with administration of the same dose of T2, we have previously shown that T2 deacetylates and inhibits SREBP-1c activity though SIRT1 activation resulting in inhibition of lipogenic gene expression (de Lange et al., 2011). The action of T2 via activation of SIRT1 has been confirmed by Shang and coworkers who showed that T2 deacetylates NF-kB in the nephropathic diabetic rat kidney (Shang et al., 2013). Regarding SREBP-1c, a different T2-mediated mechanism with a similar outcome has also been reported. Rochira and coworkers reported that T2 blocked the proteolytic cleavage of SREBP-1 in HepG2 cells without affecting its expression (Rochira et al., 2013).

In the study carried out here, T2 was administered at a dose of 25 μg/100 gBW for 1 week, a treatment which already resulted to be effective. It should be stressed that the dose of T2 should be chosen carefully in order to avoid cardiac side-effects. In the same animal model used here, an increase in heart rate was observed starting from a dose of 75 μg/100 g BW during a 3-month treatment period (Padron et al., 2014). In studies on mice higher doses were used ranging from 250 μg/100 g BW (Jonas et al., 2015) to 1,250 μg/100 gBW (Moreno et al., 2016; da Silva Teixeira et al., 2017). Because the affinity of T2 for TRs is very low but cannot be completely ruled out (Ball et al., 1997; Mendoza et al., 2013), using high doses and long treatment periods, T3-like TR/TRE-mediated transcriptional effects may come into play, including downregulation of “slow” myosin heavy chain gene transcription in muscle (de Lange et al., 2008) and in heart (Edwards et al., 1994). This is of importance in light of eventual applications in a clinical setting.

In conclusion, as a whole, this study reveals that both T2 and T3 have the capacity to modulate lipid accumulation in the liver of rats receiving a HFD; however, they act through different mechanisms. Notably, the hepatic hypolipidemic effects exerted by T2 are sustained by a decreased lipogenesis and an increased fatty acid oxidation. On the contrary, those exerted by T3 are mainly sustained by increased fatty acid oxidation (see for review Senese et al., 2014). As suggested in a previous study (Cable et al., 2009), the lipid-reducing action of T3 would have been more effective when accompanied by reduced expression of lipogenic genes, but we show here that this is not the case (*ACC* expression even being enhanced). We have previously shown that T2 is a very effective anti-lipidemic agent (de Lange et al., 2011). The short-term responses in gene expression presented here underline the observation that T2 is a strong anti-lipidemic compound, and that it may indeed be more effective with respect to T3.

## Author contributions

RS and FC performed RT-qPCR, data analysis, and wrote the manuscript; PdL, CL, LI, and ES carried out all animal studies, performed staining, and liver triglycerides estimation; MM and ALo supervised animal studies; ALa and FG supervised the study design and revised the manuscript.

### Conflict of interest statement

The authors declare that the research was conducted in the absence of any commercial or financial relationships that could be construed as a potential conflict of interest.
